# Unexpected positive intraoperative culture during supposed aseptic hip revision: Antibiotherapy is an effective and safe treatment. About 50 cases

**DOI:** 10.1051/sicotj/2026055

**Published:** 2026-10-13

**Authors:** Guillaume Auberger, Pierre-Alban Bouché, Thomas Aubert, Luc Lhotellier, Younes Kerroumi, Beate Heym, Valérie Zeller, Simon Marmor

**Affiliations:** 1 Department of Orthopedic and Trauma Surgery, Diaconesses Croix St-Simon Hospital Group 125 rue d’Avron 75020 Paris France; 2 Reference Center for Complex Osteoarticular Infection (CRIOA), Diaconesses Croix St-Simon Hospital Group 125 rue d’Avron 75020 Paris France; 3 Laboratory of Health Centers and Hospitals of Île-de-France, Diaconesses Croix St-Simon Hospital Group 125 rue d’Avron 75020 Paris France

**Keywords:** Hip prosthesis, Prosthesis infection, Unexpected positive samplings, Tsukayama, Unexpected positive culture

## Abstract

*Background*: The management of periprosthetic joint infection (PJI) in revision total hip arthroplasty (rTHA) with unsuspected positive intraoperative cultures (UPC) is not well codified. There are conflicting results in the literature. No study reported the long-term outcomes between single or both component rTHA with UPC. The aim of this study was to compare the overall survival rate of single component revision to total in case of rTHA with UPC using an institutional registry database. The reinfection-free survival rate was assessed as secondary outcomes. *Methods*: Patients were selected from a PJI institutional registry of THA between March 2003 and May 2020. Fifty rTHA with UPC were finally included in the study: 26 in the both component revision group and 24 in the single component revision group. The mean age of the patients was 64.8 years (range: 30.6; 88.2), 36% (18/50) of the patients were women and with a mean body mass index (BMI) of 27.6 Kg/m2 (range: 19.6; 39.5). Kaplan–Meier analyses were performed to compare overall survival rate between groups. *Results*: The overall survival rate in our series of rTHA with UPC was 70.0% [56.1%–87.2%] at 10 years. Fourteen re-interventions were carried out during the follow-up period (28.0%, 14/50). There was no significant difference between groups (61.4% vs. 79.3% at 10 years, respectively in both and single rTHA, *p* = 0.76). At the last follow-up, three infections were observed (6%, 3/50): 1 relapse (33.4%) and 2 new infections (66.6%). There was no significant difference between groups (83.2% vs. 100.0% at 10 years, respectively in both and single component rTHA, *p* = 0.08). *Conclusion*: Antibiotic therapy alone for UPC in a single component revision of a presumed aseptic THA is a safe and effective treatment. Therapeutic study level of evidence, level III retrospective study.

## Introduction

Prosthesis infection is the first cause of revision for total hip arthroplasty (THA) [1, 2]. Diagnosis can sometimes be challenging, as any prosthesis failure (dislocation, loosening, osteolysis, unexplained pain) can have an infectious origin. The reference test is the positivity of joint cultures by a microorganism, but other clinical, biological, and radiographic signs may suggest a low-grade infection. Therefore, the rate of infection in prostheses is probably underestimated if the signs of infection are not recognized or are absent. Revision arthroplasties, particularly hip revision procedures, are common and are associated with significant rates of postoperative infection [3–5]. Preoperative diagnosis is therefore a fundamental component of planning this surgery because of the clinical and socioeconomic consequences of peri prosthetic joint infections (PJIs) [6, 7].

Preoperative joint aspiration is thus a useful test to distinguish between mechanical prosthetic failures and prosthesis infections [8]. This procedure must be carried out under strict aseptic conditions and quickly sent to a bacteriology laboratory accustomed to handling osteoarticular samples. When possible, the cytology of the intra-articular fluid should be performed to guide whether the cause is mechanical or inflammatory [9]. In our practice, preoperative aspiration is performed before any revision of the joint prosthesis, as suggested by the literature [8]. In rare cases, there is a discrepancy between sterile preoperative samples and positive intraoperative samples. These must be interpreted cautiously and in consultation to avoid mistreating a mechanical revision as a prosthesis infection due to contamination of intraoperative samples and vice versa.

Tsukayama distinguished several types of prosthesis infections [10]. According to him, unsuspected positive cultures (UPCs) are defined by positive intraoperative cultures without preoperative evidence of infection. In this reference article, aspiration was not necessary to classify a sample as unexpectedly positive.

The treatment of these unexpectedly positive intraoperative samples is not consensual. Functional and infectious outcomes are discordant. Some believe that the infectious risk is the same as revisions for aseptic causes [11, 12], while others describe worst functional and infectious outcomes in patients with unexpectedly positive samples [13, 14]. In a recent study, Leal et al. found an infection-free survival at 5 years similar between patients who had one UPC with a new organism and those who had sterile cultures [15]. The table that summarizes the most important publishes studies is available in Appendix.

In our center, the management strategy is exclusively medical, consisting of antibiotic therapy according to modalities described subsequently, without additional surgical intervention such as synovectomy or exchange of a retained implant component in cases of partial implant revision. However, no study has compared the global survival rate of single component revision to both components (total) revision of THA with UPC.

Therefore, the primary purpose of this study was to compare the overall survival rate of single component revision (femoral stem or acetabular cup) to total revision of THA with UPC using an institutional registry database. The secondary purposes are to assess the reinfection-free survival rate and to identify risk factors of failure in revision THA (rTHA) with UPC.

## Materials and methods

All procedures performed in studies involving human participants were in accordance with the ethical standards of the national research committee and with the1964 Helsinki. This study was approved by the National Commission on Informatics and Liberty (CNIL, https://www.cnil.fr) under the reference N° 2237953. The study was registered on ClinicalTrials.gov (NCT 02801253) the 2015-09-11. The procedures used in this study adhere to the tenets of the Declaration of Helsinki. All patients gave informed consent to participate.

### Materials

#### Study design and population

Patients were selected from a PJI institutional registry of THA between March 2003 and May 2020. Inclusion criteria were patient who underwent a rTHA with UPC. Cultures were deemed “ unsuspected positive “ if at least two bacteriological cultures from intraoperative samples were positive for the same microorganism, without radiological images or clinical signs suggesting infection. Furthermore, if a preoperative aspiration was performed (41/50, 82%), it had to be negative or considered as such. During the study period, among the 945 PJI THA recorded in our registry, 50 patients (5.3%, 50/945) met our inclusion criteria: 26 in the both component revision group and 24 in the single component revision group (Figure 1).The mean age of the patients was 64.8 years (range: 30.6; 88.2), 36% (18/50) of the patients were women and with a mean body mass index (BMI) of 27.6 Kg/m^2^ (range: 19.6; 39.5). Patients had a mean follow-up of 7.6 years (range:0.3;16.5). The main cause of revision was loosening in 80.0% (40/50): 25% (10/40) femoral and acetabular loosening, 60.0% (24/40) isolated acetabular loosening and 15% (6/40) isolated femoral loosening. Demographic data for the two groups are shown in Table 1. The distribution of pathogens isolated from PJI is shown in Table 2.

**Figure 1:**
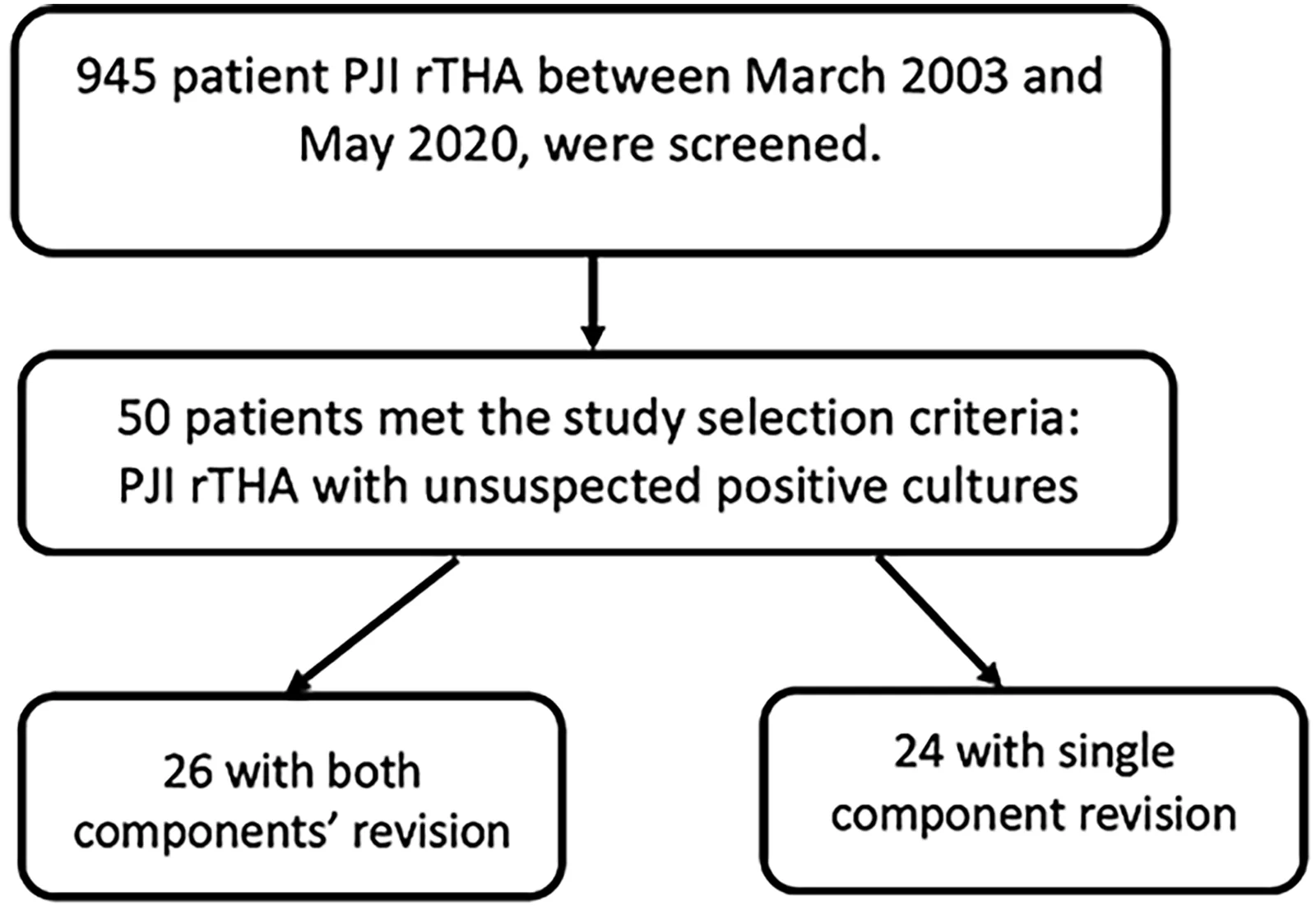
Flow chart. *rTHA: Revision Total Hip Arthroplasty.*

**Table 1:** Demographic data.

Parameters	Values	*N* = 26	Both component revision	*N* = 24	Single component revision	*p*-value
Age (years)		26	62.3 (30.6;87.7)	24	67.4 (44.5;88.2)	0.23
BMI (kg/m^2^)		26	26.8 (20.2;34.6)	24	28.4 (19.6;39.5)	0.32
Gender	Female	12	46.20%	6	25%	0.15
	Male	14	53.80%	18	75%	
Side	Right	17	65.40%	14	58.30%	0.77
	Left	9	34.60%	10	41.70%	
Number of surgeries before revision		26	2.8 (1.0;16.0)	24	1.9 (1.0;6.0)	0.25
History of PJI	No	18	0.00%	20	83.30%	0.33
	Yes	8	30.80%	4	16.70%	
Cause of revision	Cotyloiditis	1	3.80%	0	0%	0.21
	Fracture	1	3.80%	2	8.30%	
	Impingement	0	0%	3	12.50%	
	Loosening	23	88.50%	17	70.80%	
	Recurrent dislocations	1	3.80%	2	8.30%	
Follow-up after revision (years)		26	7.7 (0.3;16.5)	24	7.6 (0.3;14.3)	0.92

**Table 2:** Results of perioperative bacteriological sampling.

Pathogens	*N* = 26	Both component revision	*N* = 24	Single component revision	*p*-value
CoNS*	10	38.50%	6	25%	0.2
*Staphylococcus capitis*	1	10%	2	33.30%	
*Staphylococcus caprae*	1	10%	0	0%	
*Staphylococcus epidermidis*	3	30%	4	66.70%	
*Staphylococcus lugdunensis*	3	30%	0	0%	
SEMR*	2	20%	0	0%	
*Corynebacterium*	1	3.80%	1	4.20%	
*Cutibacterium acnes*	5	19.20%	10	41.70%	
MSSA*	0	0%	1	4.20%	
Multi bacterial	10	38.50%	5	20.80%	
Veillonella	0	0%	1	4.20%	

*CoNS : coagulase-negative staphylococci; *MSSA: Methicilin-sensitive *Staphylococcus aureus*; *SEMR: *S. epidermidis* methicilin resistant.

#### Surgical strategy and infectious management

Preoperative aspiration, when possible (41/50, 82%), was performed under local anesthesia and radiological guidance in the radiology department, at least 15 days after stopping any antibiotics, under strict aseptic conditions. The articular fluid was aspirated and then washed with physiological saline to recover two fluids, i.e., three bacteriological samples. The results of the aspiration were analyzed before the surgery. Preoperative aspiration was considered negative if all samples were negative after prolonged culture.

During the surgery, at least three samples were systematically taken and sent for bacteriological culture (synovial, acetabular, and/or femoral).

Antibiotic protocols evolved over the study period: before 2008, patients did not receive antibiotic prophylaxis but were given empirical antibiotic therapy for 2 weeks after taking bacteriological samples until the intraoperative cultures were negative. When they were positive, the antibiotic therapy was adapted to the antibiogram. Since few years, preoperative antibiotic prophylaxis has been performed (Cefazolin 2 g slow intravenous), without postoperative antibiotic therapy, according to the recommendations of the French Society of Anesthesia and Resuscitation [16].

In patients with positive intraoperative bacteriological cultures, before 2008, postoperative antibiotic therapy was adapted and prolonged. After 2008, antibiotic therapy was initiated at the time of bacteriological result knowledge. The duration and administration modalities of the antibiotic therapy (intravenous or oral) were variable and discussed in a multidisciplinary consultation meeting (depending on the germ, its antibiotic sensitivity, patient tolerance, and compliance). The antibiotic protocol is resumed in Appendix A.

All patients had clinical, radiographic, and biological monitoring during the treatment duration, followed by regular follow-up consultations to monitor the occurrence of mechanical or infectious events (3 months, 6 months, 9 months, 1 year, 2 years, then every 2 years).

### Methods

#### Outcomes

The primary endpoint of the study was the overall survival rate from single component revision (femoral stem or acetabular cup) to total revision of THA with UPC. Any re-operation (revision or not) for any reason was considered a failure. The first secondary endpoint was the occurrence of reinfection. This was defined as a recurrence of the prosthesis infection, which could be either a relapse with the same bacteria or a new prosthesis infection caused by a different bacterium. Finally, we attempted to identify risk factors for rTHA failure from any cause.

#### Statistical analysis

Frequencies and percentages were used to describe discrete variables, continuous variables were described using means and ranges (minimum and maximum). For comparative analyses, the Fischer exact test was used for percentages and the Wilcoxon test for continuous variables. Kaplan Meier analysis was performed to analyze survival rate at last follow-up. The association of risk factors with the rate of surgical revision was evaluated using univariate Cox proportional hazards regression models. Hazard ratios (HRs) are reported with the 95% CI. The significance threshold was set at 5% for a power of 80% and a type I error risk of 5%. All tests were two-sided. The R software (version 3.5.0) was used for statistical analyses.

## Results

### Primary purpose: Overall survival analysis and comparison of the two groups

There was no significant difference between these two groups (*p* = 0.76, Figure 2). The overall survival rate in our series of rTHA with UPC was 70.0% [56.1%–87.2%] at 10 years. Fourteen re-interventions were carried out during the follow-up period (28.0%, 14/50), of which 11 were for mechanical causes (78.6%, 11/14). The mechanical causes were 8 acetabular loosening (72.7%, 8/11), 2 femoral loosening (18.2%, 2/11), and 1 hematoma (9.1%, 1/11).

**Figure 2:**
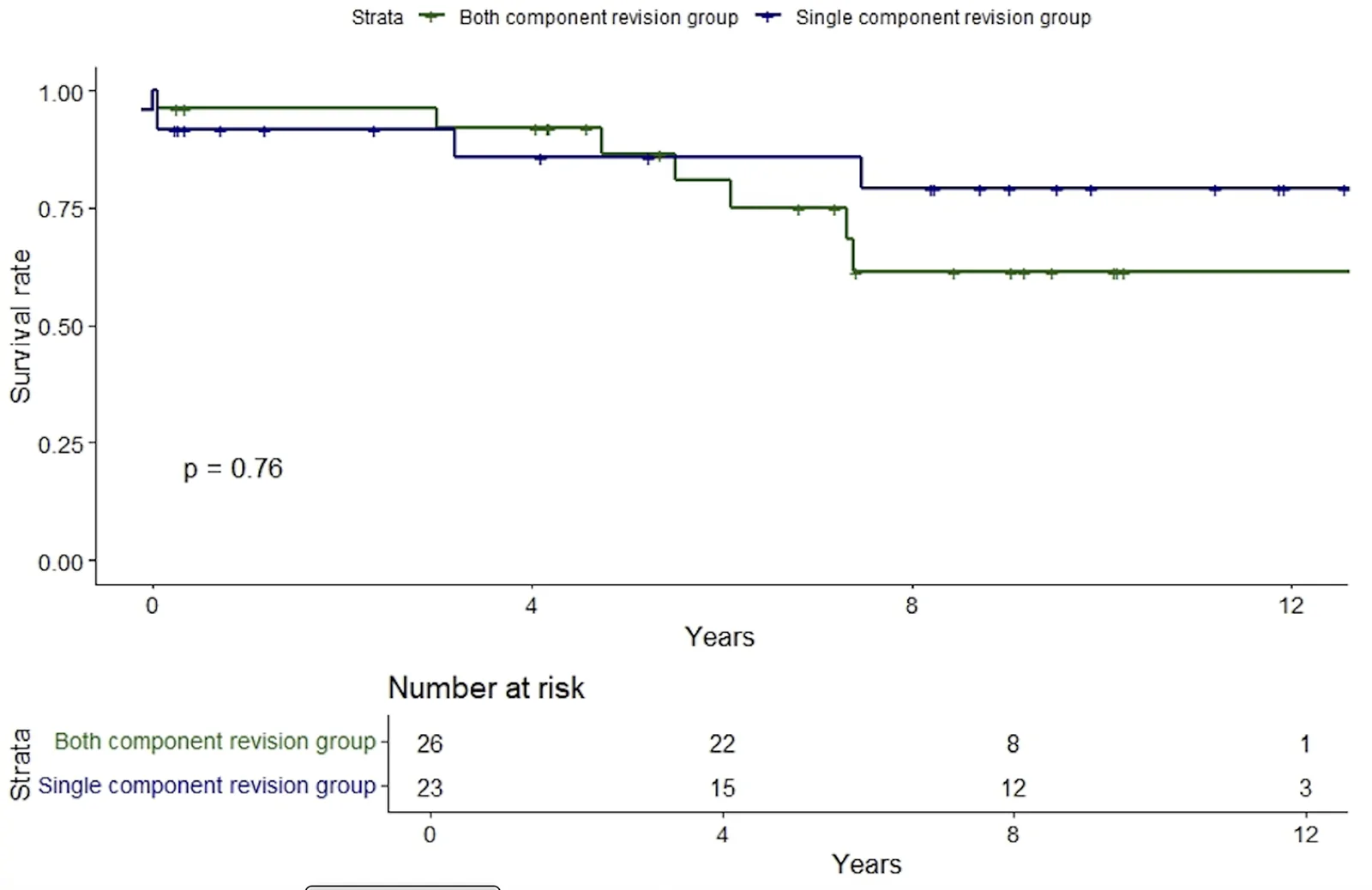
Kaplan–Meier curve of overall survival rate between both component revision group and single component revision group.

In the both component revision group, 30.8% (8/26) of THA failed: three for acetabular aseptic loosening (37.5%, 3/8), two for femoral stem aseptic loosening (25.0%, 2/8) and three for PJI (37.5%, 3/8). Overall survival rate in this group was 61.4% [42.0%–89.8%] at 10 years.

In the single component revision group, 30.8% (6/24) of THA failed: five for acetabular aseptic loosening (83.3%, 5/6) and one for hematoma (16.7%, 1/6). Overall survival rate in this group 79.3% [62.7%–100.0%] at 10 years.

### Secondary outcomes

#### Septic survival rate

At the last follow-up, three infections were observed (6%, 3/50). The mean reimplantation-reinfection interval was 4.5 [range 0.1–7.4] years. There were 1 relapse (33.4%) and 2 new infections (66.6%). The relapse was caused by an hematoma colonized by *Cutibacterium acnes* and was treated by debridement, antibiotics, and implant retention. The two new infections occurred at 6 and 7 years after the rTHA and pathogens were two *Staphylococcus epidermidis*. They were treated by a single-stage prosthesis replacement. In both component revision group, 11.5% (3/26) of rTHA had been reinfected: two new infections (2/3, 66.3%) and one relapse (1/3, 33.3%). The reinfection-free survival rate, in this group, was 96.1% [89.0%–100.0%] at 5 years and 83.2% [89.0%–100.0%] at 10 years. In the single component revision group, 0.0% (0/24) of rTHA had been reinfected. The reinfection-free survival rate, in this group, was 100.0% [100.0%–100.0%] at 5 years and at 10 years. There was no significant difference between the groups (*p* = 0.08, Figure 3).

**Figure 3:**
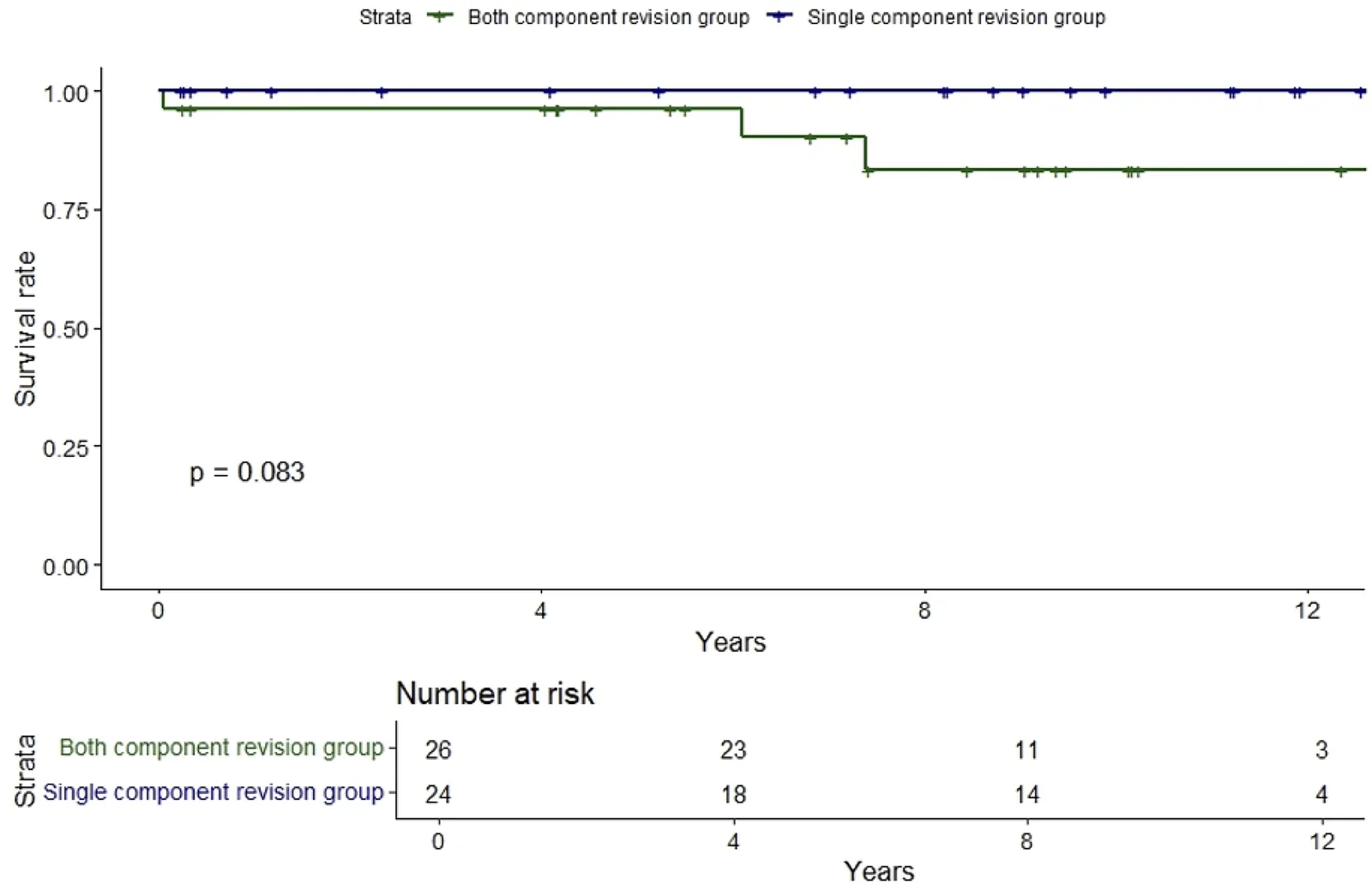
Kaplan–Meier curve of septic survival rate between both component revision group and single component revision group.

#### Risk factors associated with failure after rTHA with UPC

The variables included in the univariate analysis showed no association with the risk of failure after rTHA with UPC (Table 3). Therefore, no risk factors for failure were identified.

**Table 3:** Univariate analysis of potential risk factors for reintervention.

Characteristics	HR	95% CI	*p*-value
Age	1.04	0.99–1.11	0.11
BMI	1.07	0.94–1.22	0.30
Gender			
Woman	–	–	
Man	0.82	0.25–2.70	0.74
Delay between surgery and starting antibiotics	0.96	0.87–1.06	0.43

HR = Hazard ratio, CI = Confidence interval.

## Discussion

Unexpected positive microbiological samples are relatively common, and their management is subject to ongoing debate.

### Primary purpose: Comparison of implant retention vs. both component revision in UPC

Study reported long term results of rTHA with UPC comparing both components revisions (total revision) to single component revision. We found that there is no greater risk of failure in the single component revision group and consequently there is no need to reoperate the patient to perform a total revision. In this cohort of UPC, partial change was not a risk factor for septic failure, contrary to what is reported in the literature [13, 17].

### Secondary purpose: Infection control

While UPC may be a cause of concern for surgeons and a risk of infectious relapse with the same germ for patients, the results of our study, with only one relapse (2%), show that medical management (prolonged antibiotic therapy of 4–6 weeks and adapted to isolated germs) is reliable. Various hypotheses are proposed to explain this. First, an appropriate medical treatment by a team of infectious disease specialists trained to handle these complex cases. Secondly, the pathophysiology of UPC is still unknown, involving colonization without pathogenicity. This corroborates the bacteriology of these patients, often affected by slow-growing germs that rarely cause acute symptoms. We can make the analogy between these UPC with colonization by non-pathogenic germs like those in the urothelial tract. This non-pathogenic bacterial presence, whose risk factors (multiple operations, aseptic loosening) remain to be identified, could be specifically studied. Finally, strict preoperative criteria based on clinical, radiographic, and biological arguments and preoperative aspiration are an important component to consider. This allows the differentiation of low-grade infections from truly unexpectedly positive specimens, although the sensitivity of aspiration remains imperfect. Our good infectious outcomes are comparable to the literature, which also finds a low rate of infectious complications [18]. In a large retrospective study, some authors confirm the absence of increased infectious risk for patients with unexpectedly positive samples [19]. However, this significant cohort had many patients with only one UPC (74/109, 68%). Additionally, the infectious outcomes for patients with at least two UPC (35/109, 32%) were significantly worse. We explain our better results by a much more extensive preoperative aspiration (82% vs. 21%), allowing us to eliminate low-grade infections more effectively.

### Limitations

#### Definition of UPC and standardization by the MSIS (Musculoskeletal Infection Society)

The diagnostic consistency and number of patients, in our series, make it one of the most significant in the literature about UPC [14], and the strictest in its definition. Most of our patients (41/50, 82%) had a preoperative aspiration interpretable to differentiate a mechanical event from a prosthesis infection. The definition of UPC is not unanimous in the literature: non-systematic aspiration [20], number and quality of positive samples [11, 21]. This heterogeneity can lead to a heterogeneity of survival outcomes of the implants. Our approach aligns with the criteria set by the MSIS [22] reviewed in 2018 [23]. Unfortunately, this screening remains laborious due to its number of items, requires costly technical facilities, and depends on data often unavailable in regular practice. Moreover, its excellent sensitivity (95.5%) is not perfect, especially since its use involves intraoperative criteria, inherently too late for preoperative strategic discussion. Some studies agreed with this classification [24]. Comparing the patients in our cohort to the preoperative MSIS score could be interesting, but too much data is missing (histology, alpha defensin).

#### Uncontrolled study by an untreated comparison group

Our study is not controlled by an untreated comparison group, as has been done by other authors [14]. If the origin of these UPC remains subject to debate and can blur therapeutic strategies as suggested by the heterogeneity of care in the literature, the temptation not to perform bacteriological sampling during presumed aseptic revisions is significant [25]. This approach of not treating these patients could only be considered after a controlled prospective study comparing tailored antibiotic therapy to a no-antibiotic strategy in perfectly identified patients (without clinical, biological, or suspect preoperative aspiration arguments).

#### Monocentric and retrospective

Our study is monocentric and retrospective. However, the prospective inclusion of these patients in our historical cohort associated with the reference center for osteoarticular infections is exhaustive and particularly uniform in terms of diagnosis and surgical attitude [26]. The debate over antibiotic prophylaxis and its risk to affect intraoperative samples remains debated, but in recent years the literature has generally supported it, explaining the evolution of our practices over the duration of this study [27, 28]. Similarly, the antibiotic therapy was variable and has been reduced over the years without affecting the success of the management. Thus, we now limit the duration of postoperative antibiotic therapy to 4 weeks of oral treatment in these unexpectedly positive samples. The risk–benefit balance between not treating these patients at the risk of an infectious recurrence and over-treating them while the bacterial pressure and morbidity of prolonged antibiotic therapy are not negligible, remains pending [29]. This could be a subject for a prospective randomized study if strict preoperative analysis criteria (such as those of MSIS 2018) are respected.

### UPC: Physiopathology, challenge and clinical implication

The physiopathology of UPC remains unclear. Not really PJI, but not taking them into account is associated with an increased risk of septic complications. We can make the analogy between these UPC with colonization by non-pathogenic germs like those in the urothelial tract. This non-pathogenic bacterial presence, whose risk factors (multiple operations, aseptic loosening) remain to be identified, could be specifically studied.

Finally, strict preoperative criteria based on clinical, radiographic, and biological arguments and preoperative aspiration are an important component to consider. This allows the differentiation of low-grade infections from truly unexpectedly positive specimens, although the sensitivity of aspiration remains imperfect. Our good infectious outcomes are comparable to the literature, which also finds a low rate of infectious complications [18]. In a large retrospective study, some authors confirm the absence of increased infectious risk for patients with unexpectedly positive samples [19]. However, this significant cohort had many patients with only one UPC (74/109, 68%). Additionally, the infectious outcomes for patients with at least two UPC (35/109, 32%) were significantly worse. We explain our better results by a much more extensive preoperative aspiration (82% vs. 21%), allowing us to eliminate low-grade infections more effectively.

## Conclusion

Antibiotic therapy alone for UPC in a single component revision of a presumed aseptic THA is a safe and effective treatment. The pathophysiology of these situations remains poorly understood, and this work appears to be an interesting prerequisite before considering a prospective randomized study on the benefit or not of a delayed antibiotic treatment.

## Declaration of AI usage

Use of ChatGPT to help to ensure the traduction from French to English.

## Data Availability

Data available on request.

## Appendix A.

**Table A.** Bacteria and treatment of unexpected positive samples

Age	Bacteria	Antibiotherapy	IV Length	Total length
44	*S. epidermidis*	CFZ IV – RMP IV 3 W CFZ IV – RMP PO 3 W CFL PO – RMP PO 18 W	6	24
67	*C. acnes*	CFZ IV – RMP IV 6 W	6	6
53	*S. capitis*	CFZ IV – RMP IV 6 W	6	6
74	*S. aureus*, *S. epidermidis*, *C. acnes*	CFZ IV 7 – RMP IV 1 W CFZ PO – RMP IV 5 W FCD PO – RMP PO 6 W	6	12
72	*C. acnes*	CFZ IV – RMP IV 6 W	6	6
67	*C. acnes*, *S. epidermidis*	CFZ IV – RMP IV 4 W CDM PO 8 W	4	12
72	*C. acnes*	CFZ IV – RMP IV 6 W	6	6
71	*C. acnes*	CDM IV – RMP IV 6 W	6	6
45	*C. acnes*	CFZ IV – RMP IV 6 W CDM PO – RMP PO 6 W	6	12
60	*Corynebacterium*, *S. epidermidis*	CFZ IV – RMP IV 4 W	4	4
72	*C. acnes*	CFZ IV – RMP IV 6 W	6	6
56	*S. warneri*, *P. oryzihabitans*	CFZ IV – RMP IV 6 W	6	6
58	*S. epidermidis*	CFZ IV – RMP IV 6 W	6	6
60	*S. epidermidis*, *C. acnes, E. faecalis*	CFZ IV 7D RMP IV – VCM IV 5 W	6	6
68	*S. epidermidis*	CFZ IV – RMP IV 1.5 W VCM IV 2 W MNC PO 8 W	2	12
62	*S. epidermidis*, *C. acnes*	CFZ IV – RMP IV 4 W	4	4
57	*C. acnes*, *S. epidermidis*	CFZ IV – RMP IV 4 W	4	4
76	*Veillonella*	CDM IV – RMP IV 4 W	4	4
84	*C. acnes*	CFZ IV – RMP IV 4 W	4	4
72	*C. acnes*	CFZ IV – RMP IV 4 W	4	4
38	*C. acnes*	CFZ IV – RMP IV 4SW CFL 8 W	4	12
51	*S. epidermidis*, *S. capitis*	CDM PO – RMP PO 4 W	0	4
38	*S. lugdunensis*	CFZ IV 0.5 W FCD PO, RMP PO 4 W	0.5	4
68	*Corynebacterium*, *S. warneri*	AMX PO 4 W	0	4
50	*S. epidermidis*	CMX PO 6 W	0	6
77	*C. acnes*	CDM PO 4 W	0	4
69	*S. capitis*, *Veillonella* Sp	AMX PO 4 W	0	4
75	*C. acnes*	CDM PO 4 W	0	4
80	*S. epidermidis*	CDM PO 4 W	0	4
74	*S. aureus*, *S. epidermidis*, *S. pasteuri*	VCM IV – RMP IV 1 W CFZ IV – RMP 3 W	4	4
57	*C. acnes*	CDM PO 4 W	0	4
52	*P. harei*, *F. magna*	CFZ IV – RMP IV 4 W AMX PO 8 W	4	12
67	*S. epidermidis*	VCM IV 2 W, CFZ IV – CDM IV 4 W	6	6
67	*Corynebacterium*	VCMIV 6S, RMP IV 6 W	6	6
31	*C. acnes, C. avidum*	CFZ IV – CDM IV 1 W CDM PO 3 W	3	4
61	*S. capitis*	CFZ IV 2 W RMP PO – CDM PO 6 W	2	6
87	*C. acnes*	CDM IV puis PO*	2	12
62	*S. aureus*	CFZ – RMP IV 1.5 W CDM PO 4.5 W	1.5	6
65	*S. capitis*, *S. epidermidis*	CFZ IV – VCM IV – MNC IV 2 W CFZ – RMP IV 4 W	6	6
63	*S. caprae*	CFZ – RMP IV 6 W	6	6
64	*S. warneri*, *S. cohnii*	VCM IV – PF IV 5 W	5	5
79	*S. capitis*	CFZ IV – RMP IV 4 W	4	4
78	*C. acnes*	CDM PO 4 W	0	4
79	*S. epidermidis*	CFZ IV 1 W VCM IV – RMP IV 3 W CDM PO 4 W	4	8
59	*S. epidermidis*	CDM PO 4 W	0	4
71	*Corynebacterium*	CFZ IV 0.5 W CFZ IV – RMP IV 1.5 W CDM PO 2 W	2	4
79	*S. caprae*	CDM PO 4 W	0	4
88	*C. acnes*	CDM PO 4 W	0	4
52	*S. lugdunensis*	CFZ IV – RMP IV 4 W	4	4
56	*S. epidermidis*	CDM PO 4 W	0	4
74	*S. epidermidis*	CDM PO 4 W	0	4

## Appendix B.

**Table B.** Table with evaluation of different studies, added in annex

Studies	Patients	Diagnosis	Treatment	Septic failure
Moreira et al. [12]	9	Intraoperative positive culture ≥1	None	1 (11%)
Lara-Taranchenko et al.	8	Intraoperative positive culture ≥1	None	0
Loppini et al. [11]	60	No aspiration Inflammation in fluid +/− positive culture	Tazocillin – vancomycin and levofloxacin or ciprofloxacin 7j then etiologic antibiotherapy 3 months	1 (1.6%)
Mancheño-Losa et al. [13]	203	No aspiration Intraoperative criterion (infection possible)	Variable	17 (8.4%)
Saleh et al. [15]	103	MSIS criterion but not needed	Variable (Antibiotics or no antibiotics)	29 (28%)
Leal et al. [15]	73	1, or 2 positive cultures	No treatment	Worse outcomes if ≥2 positive culture

## References

[1] Ulrich S, Seyler TM, Bennett D, et al. (2008) Total hip arthroplasties: what are the reasons for revision? Int Orthop 32, 597–604. 10.1007/s00264-007-0364-3 PMC2551710 17443324

[2] Australian Orthopaedic Association National Joint Replacement Registry (2023) Annual Report. AOANJRR. https://aoanjrr.sahmri.com/annual-reports-2023.

[3] Schwartz BE, Piponov HI, Helder CW, et al. (2016) Revision total hip arthroplasty in the United States: national trends and in-hospital outcomes. Int Orthop (SICOT) 40, 1793–1802. 10.1007/s00264-016-3121-7 26830782

[4] Lenguerrand E, Whitehouse MR, Beswick AD, et al. (2017) Revision for prosthetic joint infection following hip arthroplasty: evidence from the National Joint Registry. Bone Joint Res 6, 391–398. 10.1302/2046-3758.66.BJR-2017-0003.R1 PMC5492333 28642256

[5] Badarudeen S, Shu AC, Ong KL, et al. (2017) Complications after revision total hip arthroplasty in the medicare population. J Arthroplast 32, 1954–1958. 10.1016/j.arth.2017.01.037 28236550

[6] Pannu TS, Villa JM, Higuera CA (2021) Diagnosis and management of infected arthroplasty. SICOT J 7, 54. 10.1051/sicotj/2021054 PMC8559719 34723789

[7] Sukeik M, Haddad FS (2019) Periprosthetic joint infections after total hip replacement: an algorithmic approach. SICOT J 5, 5. 10.1051/sicotj/2019004 PMC6394232 30816089

[8] Staphorst F, Jutte PC, Boerboom AL, et al. (2021) Should all hip and knee prosthetic joints be aspirated prior to revision surgery? Arch Orthop Trauma Surg 141, 461–468. 10.1007/s00402-021-03791-6 PMC7900020 33559723

[9] Qu X, Zhai Z, Wu C, et al. (2013) Preoperative aspiration culture for preoperative diagnosis of infection in total hip or knee arthroplasty. J Clin Microbiol 51, 3830–3834. 10.1128/JCM.01467-13 PMC3889774 23946521

[10] Tsukayama DT, Estrada R, Gustilo RB (1996) Infection after total hip arthroplasty. A study of the treatment of one hundred and six infections. J Bone Joint Surg Am 78, 512–523. 10.2106/00004623-199604000-00005 8609130

[11] Loppini M, Pisano A, Di Maio M, et al. (2021) Outcomes of patients with unexpected diagnosis of infection at total hip or total knee arthroplasty revisions. Int Orthop 45, 2791–2796. 10.1007/s00264-021-05137-8 34292384

[12] Moreira T, Lara-Taranchenko Y, Luo TD, et al. (2024) Unexpected positive cultures in conversion hip and knee arthroplasty. Int Orthop (SICOT) 48, 3049–3055. 10.1007/s00264-024-06341-y 39354162

[13] Mancheño-Losa M, Lora-Tamayo J, Fernández-Sampedro M, et al. (2021) Prognosis of unexpected positive intraoperative cultures in arthroplasty revision: a large multicenter cohort. J Infect 83, 542–549. 10.1016/j.jinf.2021.09.001 34509512

[14] Saleh A, Guirguis A, Klika AK, et al. (2014) Unexpected positive intraoperative cultures in aseptic revision arthroplasty. J Arthroplast 29, 2181–2186. 10.1016/j.arth.2014.07.010 25124809

[15] Leal J, Wu CJ, Seyler TM, et al. (2024) Unsuspected positive intra-operative cultures in aseptic revision hip arthroplasty: prevalence, management, and infection-free survivorship. J Arthroplast 1326–1334. 10.1016/j.arth.2024.10.102 39481617

[16] Verjat-Trannoy D, Arnaud I, Vagneur A, et al. (2023) Point sur les pratiques d’antibioprophylaxie préopératoire à l’échelle nationale: données, 2020–2021. Médecine et Maladies Infectieuses Formation 2, 52–57.

[17] Vargas-Reverón C, Fernández-Valencia JÁ, Martínez-Pastor JC, et al. (2025) Prevalence and impact of unexpected positive intraoperative cultures in total hip or knee revision surgery. J Arthroplast 40, 191–197. 10.1016/j.arth.2024.07.031 39053663

[18] Mangin M, Aouzal Z, Leclerc G, et al. (2023) One-stage revision hip arthroplasty for infection using primary cementless stems as first-line implants: about, 35 cases. Orthop Traumatol Surg Res 109(103642), 1–4. 10.1016/j.otsr.2023.103642 37302525

[19] Neufeld ME, Lanting BA, Shehata M, et al. (2021) Prevalence and outcomes of unexpected positive intraoperative cultures in presumed aseptic revision hip arthroplasty. J Bone Joint Surg Am 103, 1392–1401. 10.2106/JBJS.20.01559 33974575

[20] Schwarze J, Moellenbeck B, Gosheger G, et al. (2023) The role of single positive cultures in presumed aseptic total hip and knee revision surgery-a systematic review of the literature. Diagnostics (Basel) 13, 1655–1665. 10.3390/diagnostics13091655 PMC10178370 37175046

[21] Berend KR, Lombardi AV, Adams JB (2007) Unexpected positive intraoperative cultures and gram stain in revision total hip arthroplasty for presumed aseptic failure. Orthopedics 30, 1051–1053. 10.3928/01477447-20071201-17 18198778

[22] Pérez-Prieto D, Hinarejos P, Alier A, et al. (2021) Adherence to a reliable PJI diagnostic protocol minimizes unsuspected positive cultures rate. BMC Musculoskelet Disord 22(653), 1–7. 10.1186/s12891-021-04431-1 PMC8330036 34340666

[23] Praz C, Gubbiotti L, Buia G, et al. (2021) Value of the synovial C-reactive protein test in the diagnosis of total hip and knee periprosthetic joint infections: a case–control study. Orthop Traumatol Surg Res 107(102903), 398–405. 10.1016/j.otsr.2021.102903 33775885

[24] Altsitzioglou P, Avgerinos K, Karampikas V, et al. (2024) Point of care testing for the diagnosis of periprosthetic joint infections: a review. SICOT J 10, 24–36. 10.1051/sicotj/2024019 PMC11160401 38847648

[25] Mainard N, Saab M, Dartus J, et al. (2022) The benefits of systematic intraoperative sampling during lower limb arthroplasties due to sequelae from prior osteoarticular infections: a retrospective study of, 92 cases. Orthop Traumatol Surg Res 108(103189), 1–6. 10.1016/j.otsr.2021.103189 34933132

[26] Ferry T, Seng P, Mainard D, et al. (2019) The CRIOAc healthcare network in France: a nationwide Health Ministry program to improve the management of bone and joint infection. Orthop Traumatol Surg Res 105, 185–190. 10.1016/j.otsr.2018.09.016 30413338

[27] Bedenčič K, Kavčič M, Faganeli N, et al. (2016) Does preoperative antimicrobial prophylaxis influence the diagnostic potential of periprosthetic tissues in hip or knee infections? Clin Orthop Relat Res 474, 258–264. 10.1007/s11999-015-4486-4 PMC4686528 26253269

[28] Pérez-Prieto D, Portillo ME, Puig-Verdié L, et al. (2016) Preoperative antibiotic prophylaxis in prosthetic joint infections: not a concern for intraoperative cultures. Diagn Microbiol Infect Dis 86, 442–445. 10.1016/j.diagmicrobio.2016.09.014 27745737

[29] Tsukayama DT, Wicklund B, Gustilo RB (1991) Suppressive antibiotic therapy in chronic prosthetic joint infections. Orthopedics 14, 841–844. 10.3928/0147-7447-19910801-07 1923965

